# University Credits as a Measure of Teachers’ Pre-service and In-Service Training: A Longitudinal Approach Using Swedish Data

**DOI:** 10.3389/fpsyg.2021.709624

**Published:** 2022-01-11

**Authors:** Stefan Johansson, Åse Hansson, Tarja Alatalo

**Affiliations:** ^1^Department of Education and Special Education, University of Gothenburg, Gothenburg, Sweden; ^2^School of Teacher Education, Dalarna University, Falun, Sweden

**Keywords:** teacher knowledge, pedagogical content knowledge, university credits, GPA, longitudinal studies, pre-service training, in-service training, professional development

## Abstract

In this study, we accessed information about the university credits of all teachers born after 1971 in Sweden as a means of ascertaining the development of their subject knowledge. We examined the university credits they earned during pre-service and in-service training. Data comes from registers Gothenburg Educational Longitudinal Database (GOLD) and the teacher register. We linked GOLD to the teacher register in order to describe the knowledge development of teachers in compulsory school 1998–2014. Special focus was on Swedish language and mathematics. Multiple regression and multilevel growth modeling were used as our main methods. Results show an increase in pre-service credits during the time period and more credits in Swedish language than in mathematics. To analyze teachers’ in-service training, we followed the development of their university credits over time. Teachers with higher prerequisites in terms of grade point average tended to gain more credits in-service. The study included discussions on ideas and the implications for future research.

## Introduction

In recent decades, teacher quality has shown itself to be a key issue for schools that significantly affects student achievement (e.g., Nye et al., 2004; Rockoff, 2004; Rivkin et al., 2005; Darling-Hammond, 2016). Despite numerous studies demonstrating the substantial effects of teacher quality, there is little agreement as to how to conceptualize teacher quality or as to which characteristics are effective (e.g., Hanushek and Rivkin, 2010). Darling-Hammond (2021), however, noted some commonalities for successful school-systems. These were characterized by, for example, high-performing student teachers and a master’s degree for every teacher, as well as clearly framed standards of what knowledge every teacher should have.

Standards for teacher knowledge largely depart from Shulman’s (1986, 1987) work. Shulman developed the well-known theoretical distinction between teachers’ content knowledge (CK) and pedagogical content knowledge (PCK). CK refers to the current knowledge within a subject-domain, while PCK comprises ways of representing and formulating CK that makes it comprehensible to students. PCK represents the blend of content and pedagogy into an understanding of how particular topics and issues are organized, represented, and adapted to the diverse interests and abilities of learners. The necessity of deep subject knowledge has been proposed as foundational to effective teaching. Darling-Hammond (2016), for example, argue that teachers’ CK and PCK interact and that they together determine teacher effectiveness. Moreover, teachers need generic competencies concerning, for example, classroom management, lesson planning, and assessment, which is often referred to as general pedagogical knowledge (GPK).

Effective teachers are thus characterized by a complex composite of knowledge and skills, and it is difficult to delineate the components of CK and PCK empirically. Hill et al. (2005), for example, suggested that the CK and PCK in mathematics of primary school teachers could be merged into one unit of knowledge: mathematical knowledge for teaching (MKT). Others have found that CK and PCK are separable and unique dimensions, be they nonetheless correlated (Phelps and Schilling, 2004; Krauss et al., 2008; Baumert et al., 2010). Baumert et al. (2010) showed that despite the high correlation between CK and PCK, CK had lower predictive power for student progress than did PCK. CK was, however, an important prerequisite for PCK. This was confirmed in a similar study by Callingham et al. (2016). Moreover, teachers’ CK has been found to relate to the appropriateness in teachers’ feedback to students’ mathematical ideas and to the accuracy of teachers’ mathematical language (e.g., Spillane, 2000; Hill et al., 2008), and also to their implementation of mathematics curriculum materials (e.g., Manouchehri and Goodman, 2000; Sherin, 2002). Mathematics CK is also a significant predictor of pre-school teachers’ ability to perceive learning situations and to plan educational actions that foster learning (Dunekacke et al., 2015). Teachers’ PCK is shown to relate to their instructional quality (e.g., Kunter et al., 2013), and, for example, to knowledge of students’ thinking about mathematics (e.g., Lubinski, 1993).

For the case of reading, primary school students’ learning is improving as a result of the training teachers receive pre-service and that prepares them for the profession and the teaching of primary school students (Croninger et al., 2007). Having a master’s degree has not shown consistent results for student achievement (Rockoff, 2004; Rivkin et al., 2005; Leigh, 2010). For mathematics in lower-secondary school, however, a master’s degree increases the ability of a teacher to boost student achievement (Harris and Sass, 2011), as is also the case for secondary school mathematics teachers (Lachner and Nückles, 2016). Croninger et al. (2007) also show the importance of contextual effects, such as collective expertise at the school level in literacy, which could develop stronger curricular programmes and provide pedagogical support to less qualified colleagues, boosting school-wide disciplinary cognitive gains. As regards mathematics, Seidel and Shavelson (2007) showed in a meta-analysis that the greatest effects of teaching derive from disciplinary components of mathematics teaching obtained during pre-service teacher training, not from the way teaching is organized. Indeed, teachers’ course work relating to mathematics is a factor often believed to have positive effects on student achievement (Harris and Sass, 2011). For language teachers, course work in reading (e.g., Clotfelter et al., 2007) and language (e.g., Adams and Lowey, 2007; Hart et al., 2009; Lamb, 2010) is also crucial. However, Monk (1994) showed that the effects of teachers’ disciplinary course work in mathematics and science are less significant than additional training in pedagogy.

Besides the pre-service training, teachers’ CK and PCK can be developed by in-service training (Kleickmann et al., 2013). Several studies suggest that these aspects of teacher knowledge can improve by way of further training (e.g., Copur-Gencturk, 2015; Kennedy, 2016). To invest in professional development activities may be beneficial for student achievement but also for teachers’ self-efficacy and job satisfaction (Boeskens et al., 2020). How to design effective in-service training is, however, not clear. To be effective, research has suggested that the training should focus on CK and be designed as collective and intense participation programmes (Kennedy, 2016). If the programmes focus only teachers’ pedagogical knowledge, however, research has documented null effects on student achievement (e.g., Dash et al., 2012). As with mathematics, teachers’ knowledge about reading improves through intensive, extended programmes of professional development in literacy (McCutchen et al., 2002; Moats and Foorman, 2003; Carlisle et al., 2011). While several studies show the positive effects of further training, it should be noted that research is inconsistent about how to best develop teachers’ knowledge. Some researchers have proposed that the pedagogical part of teacher knowledge is difficult to develop through education and that these skills are better developed by way of work experience (e.g., Hanushek, 2011; Chingos and Peterson, 2013).

Naturally, there are other factors besides professional development programmes that can influence the effectiveness and likelihood to participate in in-service training. Teachers’ background, for example, their prior knowledge and teaching experience could influence both the outcome and amount of in-service training teachers enroll in. The extent to which teachers undergo in-service training may also depend on if it is voluntarily or not—some initiatives are school-based and mandatory, whereas others are teachers’ own initiatives. The self-determination theory (SDT) may be useful in explaining both why individuals enroll in teacher training as well as why they take in-service training. SDT concerns the motives that steer individual’s choices and describes how and why some individuals are more proactive and engaged (Ryan and Deci, 2000). Some of the teachers are likely more self-determined than others; they are driven by intrinsic motivation and strive for competence and autonomy, which indeed are central parts of the SDT. The role of teachers’ prior knowledge might influence both the likelihood of enrolling in in-service training and its outcome—especially if training is taken on voluntary basis. In the best of worlds, teachers with less knowledge would enroll in further training to a higher extent than teachers with more knowledge. In that way, successful in-service training could compensate for some teachers’ lower prior knowledge and weaker subject knowledge. This may be a particularly pertinent issue in Sweden where the prior knowledge of new teachers is shown to have decreased in recent years (Bertilsson, 2014; Alatalo et al., 2021). Since the mid-1990’s, Sweden experienced extensive school reforms: for example, new curricula, new teacher education, and decentralized governance of schools.

### Aim

Teachers develop their knowledge in two major ways: By pre-service training and in-service activities including reflection on teaching experiences as well as professional development which could both be out of school and/or school embedded. Pre-service and in-service activities develops teacher quality and this in turn affects student achievement. However, it has been difficult to establish any significant effects of length of pre-service training (Rivkin et al., 2005) or in-service training (Kennedy, 2016). This is due to a number of reasons, for example lack of precision in how pre-service and in-service activities were operationalized. In this study, we exploit a unique dataset comprising every university credit that teachers earned during both their pre-service and in-service years. We were able to examine every course that teachers took and when they took it, and we were also able to categorize these courses into mathematics and Swedish language domains. However, it was beyond the scope of this study to distinguish between CK and PCK aspects due to the large variation in terminology.

The main aim of the study is rather explorative: We investigate the possibilities to operationalize a more precise measure of teachers’ pre-service and in-service training than has been provided in much of the previous research. We will shed light on the development of teachers’ pre-service and in-service training in terms of their university credits in Sweden from 1998 to 2014, with special focus on mathematics and literacy. We hypothesize a non-linear increase for in-service training because we anticipate that teachers do not participate in these activities the first years in the profession to high extent. We also hypothesize that teachers with higher own final grades (GPA score) enroll to higher degree in further training and that no compensatory function can be observed from in-service training. Furthermore, we investigate differences relating to the Grade taught: primary (Grades 1–6)/secondary (Grades 7–9). We begin our investigation with determining the number of pre-service credits for teachers during the period 1998–2014.

Specific research questions were:

(1) To what extent did Swedish teachers enroll in university based pre-service and in-service training during 1998–2014?
(2) How did teachers’ own GPA influence the likelihood of enrolling in in-service training?
(3) Were there any differences in the amount of in-service training for different Grade levels?

## Materials and Methods

To answer our research questions, we used data from the Swedish teacher register provided by Statistics Sweden. In this data, the complete population of teachers in Swedish schools is present, including detailed information about their position (e.g., the disciplinary subject they teach), their teacher education, and their certification status. In this study, we linked data from The Gothenburg Educational Longitudinal Database (GOLD), which stores other information about all individuals born after 1971 in the teacher register data. Data from 1998 to 2014 was used in the current study. The credits for the university courses were first registered in the GOLD database in 1993–1994, but relatively few of the GOLD teachers were at that time enrolled in higher education. Since there was also a lag in the registration of credits, we selected 1998 as a starting point for analyses using university credits. The larger research project in which our study was conducted had available data on university credits up to 2014. The registers are presented below in more detail.

### The Teacher Register and GOLD

The teacher register forms part of the national follow-up system for the school sector of the Swedish National Agency for Education. Its purpose is to provide a comprehensive picture of school activities as well as support for follow-up and evaluation at the national and regional level. Information is collected annually for all school staff with educational duties (for example, teachers, assistant teachers, pre-school teachers, recreation instructors, school leaders, and study and career counsellors). In the current study we make use of those having a position as teacher. The information about teachers in the register is most often provided by the principal. Data has been collected since the late 1970s, and the structure and variables of the register have changed over the years.

A unique component of the teacher register data is that it is stored by personal identification number, which facilitates a link between the teacher register and the national database GOLD, which also uses the personal identification number system. GOLD contains data for the complete population in Sweden born after 1971. Data on individuals is stored from the year a person is 16 years old and is updated yearly. As such, both the teacher register and GOLD contain longitudinal information at the individual level, where certain characteristics are fixed whereas others vary. For each individual there is comprehensive information about family background, school achievement, employment, and income, among other things. When we merge GOLD with teacher register data, we are able to investigate teachers’ pre-service and in-service training in a detailed manner. Since GOLD includes individuals born after 1971, we did not analyze all teachers in compulsory school. The number of GOLD teachers increases over time. For 1998, we mainly looked at young teachers because those born in 1972 are 26 years old in 1998. In 1998, the data on some 5,700 teachers was available in GOLD. In 2014, we analyzed approximately 50% of all teachers in Sweden, at which time the number of GOLD teachers was about 42000.

#### Variables

Our analysis focuses on disciplinary course work as the variable of interest. This output variable is described in the following. In addition, we used several covariates to shed light on development over time for different teacher groups. These include: subject (focus on mathematics/Swedish language), Grade [primary (Grades 1–6)/secondary (Grades 7–9)], teachers’ own final grades (GPA) from compulsory school (Grade 9). Because grades have been subject to grade inflation, every individual was assigned a percentile transformed grade. Percentiles were computed for each cohort separately, so for each cohort the GPA had a mean of around 50 and a SD of around 28.

We use the total number of teachers’ university credits each year as our outcome variable to measure length of pre-service training. A national credit system is used by Swedish universities to show the scope of a course or study program where one week of full-time studies (40 h) corresponds to 1.5 higher education credit. Each semester is 20 weeks long, during which a student is expected to take 30 credits’ worth of courses. One Swedish credit is equal to one ECTS credit. The European Community Course Credit Transfer System, was developed by the Commission of the European Communities (ETCS) in order to provide common procedures to guarantee academic recognition of studies abroad. It provides a way of measuring and comparing learning achievements, and transferring them from one institution to another.

Gothenburg Educational Longitudinal Database includes every university course taken by individuals born after 1971. We used the total number of credits held by teachers as well as their credits in mathematics and Swedish language. As we were particularly interested in courses concerning mathematics and Swedish language, we dummy-coded courses that indicated CK or PCK in mathematics and Swedish language and used these as explanatory variables in the analyses.

### Analytical Methods

This study relies on several statistical methods. In order to describe the general trends of teachers’ pre-service training, we used multiple regression and descriptive analysis methods. We studied the complete GOLD population of teachers as well as the newly recruited teachers each year.

To gain insight into the development of teachers’ in-service training, we carried out multilevel growth modeling using the mixed model for repeated measures provided in SPSS 26.0. The design implied that measurements were taken for different individuals at different points in time (teachers signed up for in-service training at different occasions during their career). Such complex designs can be effectively examined using multilevel modeling techniques (Hox, 2002). The basic model for investigating in-service trajectories is presented in Equation 1. The longitudinal analyses allowed us to investigate more deeply how in-service training changed over time as well as if the credit change was the same for teachers with different characteristics. All analyses were carried out using SPSS 26.0.

## Results

The research questions concern teachers’ university based in-service training and what influences the development of the number of credits. Firstly, we will explore the development of credits taken pre-service in order to provide a comprehensive picture of how the length of teachers’ education has varied across the last decades.

### Pre-service Course Work

To examine the development of credits in detail, we conducted regression analysis. Because the number of GOLD teachers and the length of teacher education has increased over time, we controlled for time and teachers’ age in the regression. We focused on teachers who had an education, either complete or in part. Thereby we selected teachers who had earned at least 60 credits before being employed as teachers. This makes the comparison of total number of credits less sensitive to variation in the proportion of uncertified teachers, which has varied greatly across years. Uncertified teachers that are employed on temporary basis sometimes do not hold any credits from the university.

We began by including only the year in a simple regression in Model 1. The results presented in Table 1 showed an increase in the number of credits over time, which is not surprising given that teacher education became somewhat longer for teachers who underwent training after 2001. We also included a quadratic time variable in Model 2 to account for any curvilinear trends. Introducing a quadratic year term rendered a model with higher explained variance (R^2^), and it was therefore retained in further models. The quadratic term suggests that the increase in credits deaccelerates and flattens out by the end of the time period. We noted that when year was kept constant, results showed older teachers to have somewhat fewer credits. In Model 3, the number of credits to decrease by 0.5 for teachers’ age. However, the number of credits increased over time, regardless of teachers’ age. In other words, a 26-year-old in 2014 had more credits than a 26-year-old in 1998. Furthermore, we introduced teachers’ GPA and the grade they are working in, primary or secondary school, as dummy variables (primary/secondary). Model 4 demonstrates that secondary school teachers have on average 50 credits more than primary school teachers. Secondary school teachers have generally higher GPA than primary school teachers, one reason for this being higher demands for admission to teacher education for secondary school teachers. However, keeping the grades constant, we noted a higher production of credits for those who had a high GPA. In the final step, we introduced three dummies in the model for GPA. We divided the continuous GPA-variable into four categories based on the standard deviation. The reference category was those with a low GPA, i.e., more than 1SD below the GOLD teacher population mean. The results suggest that for those who have more than 1SD above the average GPA (GPA4), the number of credits is nearly 11 points higher, regardless of the grade taught.

**TABLE 1:** Linear regression for the development of teachers’ university credits.

Model		Unstandardized coefficients		Standardized coefficients	*t*	*p*
		B	SE	Beta		
1	Intercept	225.452	0.292		770.825	0.000
	Year	2.667	0.028	0.183	96.861	0.000
2	Intercept	208.632	0.472		442.281	0.000
	Year	7.759	0.116	0.533	67.119	0.000
	Year _quad	–0.287	0.006	–0.360	–45.342	0.000
3	Intercept	221.191	1.009		219.243	0.000
	Year	8.087	0.118	0.556	68.602	0.000
	Year _quad	–0.285	0.006	–0.357	–44.983	0.000
	Age	–0.506	0.036	–0.037	–14.081	0.000
4	Intercept	208.204	0.930		223.977	0.000
	Year	7.291	0.108	0.501	67.221	0.000
	Year _quad	–0.251	0.006	–0.316	–43.180	0.000
	Age	–0.581	0.033	–0.042	–17.577	0.000
	Secondary (1)	50.937	0.229	0.385	221.952	0.000
5	Intercept	200.849	0.973		206.503	0.000
	Year	7.566	0.109	0.521	69.538	0.000
	Year _quad	–0.260	0.006	–0.327	–44.615	0.000
	Age	–0.615	0.033	–0.045	–18.621	0.000
	Secondary (1)	50.574	0.230	0.382	220.262	0.000
	GPA4	10.760	0.389	0.065	27.683	0.000
	GPA3	8.010	0.337	0.061	23.800	0.000
	GPA2	6.208	0.363	0.042	17.112	0.000

**FIGURE 1:**
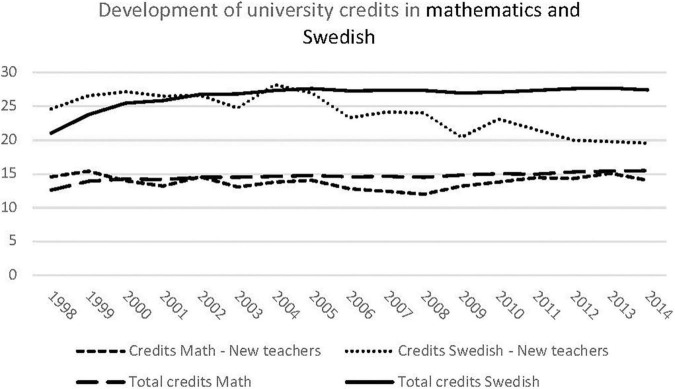
Development of university credits in mathematics and Swedish language.

Moreover, we noted that Swedish language teachers have more credits than mathematics teachers, which is interesting considering the increased focus on measures to develop mathematics teaching in recent years. We therefore explored the increase in the number of credits in mathematics and Swedish language by way of a detailed categorization of credits into mathematics and Swedish language. Figure 1 sheds light on the number of credits in mathematics and Swedish language, respectively. It may be noted that the proportion of credits is fairly low for both subjects. This is because many teachers in the data set teach subjects other than mathematics and Swedish, and thus naturally do not study these subjects in their pre-service training. More studies are generally devoted to Swedish language than mathematics, possibly because more subjects include training in Swedish than in mathematics. However, for the new teachers, the number of mathematics credits has increased in recent years, while the number of Swedish credits has decreased.

### In-Service Course Work

In the following, we will analyze how in-service training developed between 1998 and 2014 by selecting a group of teachers that worked for several years.

We considered university credits that teachers earned after their teacher certification as *in-service training*. In the analyses, we selected teachers who were certified and who received their teaching certification in 1998, meaning they were typically aged between 22 and 26 when they started their teaching career, and who were born between 1972 and 1976. This allows us to study a homogeneous group longitudinally from the years 1998–2014. We followed this graduation cohort over time and studied the development of in-service training with special focus on the cohort’s prior knowledge and differences due to their teacher specialization (grades and subjects taught). We investigated in-service training for about 1,500 teachers that at least had 5 years of experience. In total we had about 17,000 observations. The design was complex because not all teachers started to work at the exact same year and they took in-service training at different occasions during their career.

To shed light on teachers’ in-service trajectories, we used SPSS Mixed to perform longitudinal analyses by means of a two-level growth model. At level 1, individuals’ successive measurements over time were defined by an individual growth trajectory and random error. We used each teacher’s number of credits the year after graduation as our dependent variable. Credits accumulated over time for those who enroll in further training. At the second level, differences in trajectories between groups of individuals can be explored. In a first step, we defined the shape of the teachers’ growth trajectories by determining whether the initial intercept and random time slope varied across individuals. Next, since the intercept and growth rates varied across individuals, we introduced a set of predictors at level 2 (Grades, GPA, subject taught) to explain the differences in teachers’ initial number of credits and their growth trajectories. To investigate this, we constructed cross-level interactions that involved the effects of level 2 variables on level 1 coefficients. Level 2 variables were Grades (primary/secondary), GPA, and subject—variables that do not vary by individual but across individuals. The level 1 slope coefficient was the growth of teachers’ yearly earning of credits. Prior to the analysis, we recoded the year variable to match the starting year and the executive years for each teacher (0 for year 1998 if teachers started to work in 1998). We also defined a quadratic time variable (time × time) to capture any changes (acceleration or deceleration) in the rate of change that might occur over the time period. In Equation 1, the basic model without level 2 predictors is presented:


(1) $$Y_{t\,i}=\pi_{0\,i}+\pi_{1\,i}+\pi_{2\,i}+\varepsilon_{t\,i},$$

where,


$$\pi_{0\,i}=\beta_{00}+r_{0\,i},$$

$$\pi_{1\,i}=\beta_{10}+r_{1\,i},$$

$$\pi_{2\,i}=\beta_{20}+r_{2\,i},$$

Where one individuals *i*’s credits Y at time *t* is predicted by an intercept, π_0_*_*i*_*, and a linear growth slope, π_1_*_*i*_* as well as a quadratic growth slope π_2_*_*i*_* at level 1.The subscript *i* indicates that the model estimates a separate intercept and a separate linear growth slope for each person in the sample. Therefore, each teacher in the sample can have a unique linear growth rate and a unique intercept. Between teachers we can investigate three within-individual coefficients as randomly varying where *r* is the residual for each equation. As teachers’ in-service develop in different ways we keep all the random terms (*r*) in the equation. Through the substitution of the equations above, we can arrive at the single-equation model without level 2 predictors.


(2) $$Y_{t\,i}=\beta_{00}+\beta_{10}+{\left(y\,e\,a\,r\right)}_{t\,i}+\beta_{20}\,{\left(y\,e\,a\,r\,\_\,q\,u\,a\,d\right)}_{t\,i}+r_{0\,i}+r_{1\,i}+r_{2\,i}+\varepsilon_{t\,i}$$

In order to more deeply explore the growth trajectories for different teachers we constructed a series of two-level growth models. We introduced GPA and grade-level (primary/secondary). Disciplinary subject had no relationship to in-service credits and results for this model were therefore not reported. To explore any differences in growth rates, we computed interaction terms. The final model including level 2 predictors is presented in Equation 3.


$$Y_{t\,i}=\beta_{00}+\beta_{10}+{\left(y\,e\,a\,r\right)}_{t\,i}+\beta_{20}\,{\left(y\,e\,a\,r\,\_\,q\,u\,a\,d\right)}_{t\,i}+\beta_{01}\,{\left(G\,P\,A\right)}_{t\,i}$$

$$+\beta_{02}\,{\left(S\,e\,c\,o\,n\,d\,a\,r\,y\right)}_{t\,i}+\beta_{11}\,{\left(y\,e\,a\,r*G\,P\,A\right)}_{i}+\beta_{12}\,{\left(y\,e\,a\,r*s\,e\,c\,o\,n\,d\,a\,r\,y\right)}_{i}$$

$$+\beta_{21}\,{\left(y\,e\,a\,r\,\_\,q\,u\,a\,d*G\,P\,A\right)}_{i}+\beta_{22}\,{\left(y\,e\,a\,r\,\_\,q\,u\,a\,d*s\,e\,c\,o\,n\,d\,a\,r\,y\right)}_{i}\,r_{0\,i}+r_{1\,i}$$

(3) $$+r_{2\,i}+\varepsilon_{t\,i}$$

The results are presented in Table 2. To assess the fit for the models we ran we compared AIC and BIC values. From Model 1 we note that the average earned credits at their year 0 is 238. The yearly increase β_10_ is on average about 2.2 credits, however, when the quadratic term β_20_ is added in Model 2, model fit improves and the results suggests a significant acceleration over time. This is reasonable as teachers do not enroll in further training just after graduation but rather after some few years. The random part of the model indicates significant variability in both intercept and growth rate. The numbers of the random part of the model are not straightforward to interpret, however, it should be noted that variability is highly significant in all models although it decreases slightly when we add predictors.

**TABLE 2:** Parameter estimates for the fourth growth models.

		Parameter	Model 1	Model 2	Model 3	Model 4
Fixed effects, initial status π_0*i*_	Intercept	β_00_	237.92** (1.26)	238.50** (1.25)	211.50** (6.55)	212.60** (5.73)
	Slope	β_01_ (GPA)			0.34** (0.08)	0.16* (0.07)
		β_02_ (Secondary)				53.49** (2.54)
Rate of change, π_1*i*_(Year)	Intercept	β_10_ (Year)	2.20** (0.11)	1.83** (0.13)	0.41 (0.70)	−0.40 (0.70)
	Slope	β_11_ (GPA × Year)			0.02* (0.01)	0.02* (0.01)
	Slope	β_12_ (Secondary × Year)				−0.21 (0.31)
Rate of change, π_2*i*_(Year_quad)	Intercept	β_20_ (Year_quad)		0.05** (0.01)	0.12* (0.06)	0.12* (0.06)
	Slope	β_21_ (GPA × Year_quad)			−0.001 (0.001)	−0.001 (0.001)
	Slope	β_22_ (Secondary × Year_quad)				0.02 (0.02)
Variance		Var(r_0i_) = T_00_	2,295.82 (85.81)	2,243.97 (84.09)	2,182.39 (82.28)	1,664.64 (62.99)
		Var(r_1i_) = T_11_	17.15 (0.70)	20.57 (0.96)	20.09 (0.94)	20.15 (0.94)
		Var(r_2i_) = T_22_		0.11 (0.007)	0.11 (0.007)	0.11 (0.007)
Goodness-of-fit		AIC	129,904.84	128,163.37	126,342.03	125,958.39
		BIC	130,036.41	128,302.67	126,481.09	126,097.45
		Deviance	129,870.84	128,127.37	126,306.03	125,922.39
		Parameters	20	21	23	27

*Unstandardized coefficients, standard errors are within parenthesis.*

**p < 0.001.

*p < 0.05.

**FIGURE 2:**
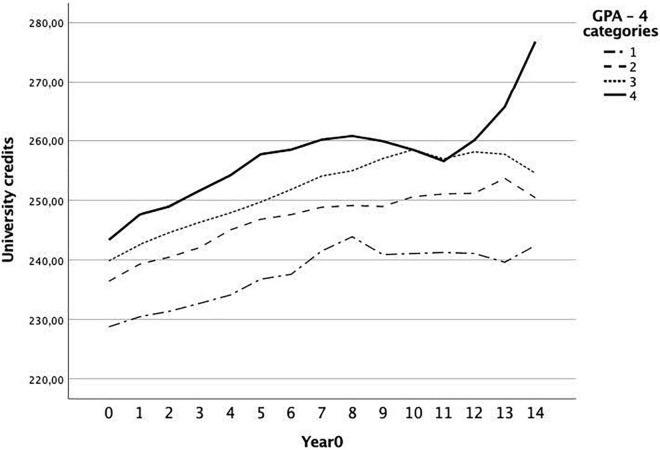
Development of university credits in-service for teachers with different GPA levels.

Furthermore, in Model 3, we introduce teachers’ own GPA to the model. GPA has a positive significant effect on number of credits (β_01_ = 0.34) suggesting that teachers with higher GPA have more credits. The intercept (β_00_ = 211.50) decreased substantially as teachers with a GPA of 0 have a lower predicted number of credits. When we keep the parameters in Model 3 constant, the average yearly growth of credits is no longer significant, however, this might be explained by the interaction term between year and GPA (β_11_ = 0.02). The interaction suggests that yearly growth rate differ between teachers with a different GPA levels. Teachers with higher GPA tend to make slightly more growth per year as compared with their counterparts with lower GPA. When we graphed (see Figure 2) the development for four GPA categories, we observed a sharp increase of credits for teachers that were in the highest GPA category.

In Model 4, we introduced grade-level. The significant estimate β_02_ = 53.49 suggests that teachers at the secondary level have an estimated starting level of their credits that is more than 50 points higher than lower secondary teachers, holding GPA constant. The interaction term is not significant suggesting there were no noteworthy differences in growth per year for teachers at different grades.

It should be noted that teachers enroll in in-service training to a higher extent than what is shown in these findings as we only focused on credits earned at university. Nevertheless, we find no indication that in-service training would work compensatorily in that teachers with lower prerequisites complete more in-service training than do their counterparts with a higher GPA: in fact, the results show quite the opposite. The growth trajectories seem fairly similar for the different groups of teachers, even though teachers with the higher GPA tended to complete in-service training to a somewhat higher extent.

## Discussion

The aim of the study was to investigate teachers’ pre-service and in-service training in terms of university credits in Sweden 1998–2014. There was an increase in the number of credits achieved pre-service by teachers in Swedish schools, but we observed a decrease in more recent years. This might have to do with the fact that newly-recruited teachers in recent years begin working before they are fully certified, i.e., they do not have all credits but a fair amount.

Some differences as regards our examination of the number of credits in mathematics and Swedish language we shown. The fact the number of credits pre-service in mathematics has increased in recent years compared with Swedish language may be due to the recent increase in focus on mathematics. Declining results in international mathematics assessments combined with a lack of mathematics teachers may have driven the reforms. While we see less focus on language over time, it is possible that the courses in mathematics include didactical aspects that strongly relate to language. Several studies show that teachers’ ability to teach mathematics depends highly on their literacy skills (Adams and Lowey, 2007; Lamb, 2010). In light of the increasing number of immigrant students in Swedish schools, it is crucial that teachers have a large linguistic repertoire so that they can provide effective instruction to those who need it the most.

As expected, we found that teachers at secondary school have more credits than those at primary school. This pattern is constant over time and is a function of the length of education. Regardless, however, of grade, our study suggests that teachers with a higher GPA have more credits. This pattern is constant over time and holds true for both pre-service and in-service training.

International evidence suggests that teachers spend about 11 days per year engaged in professional development activities like courses, workshops and in-service training (Sellen, 2016). However, in the present study, we focused on in-service training in terms of credits achieved at university level. Boeskens et al. (2020) describe an analytical framework for teachers’ professional development that might be useful for guiding teachers’ in-service training as conceptualized in our study. They describe different dimensions of high-quality in-service training where the *motivation* dimension is particularly relevant for the present study to consider. What shapes teachers’ motivation to engage in in-service training at university? To undergo university courses is reasonably an active choice by a motivated teacher rather than an incentive provided by the school principal—who commonly introduce workshop interventions for all teachers at the school. The result that more high-achieving teachers undergo more in-service training might be explained along the lines of the SDT (e.g., Ryan and Deci, 2000). SDT concerns the motives that steer individual’s choices and describes how and why some individuals are more proactive and engaged. Some of the teachers are likely more self-determined that others; they are driven by intrinsic motivation and strive for competence and autonomy, which indeed are central parts of the SDT. From this point of view, it is reasonable that the teachers who got higher grades in the end of compulsory school continue to strive to improve and master the challenges they face, for example, undergo in-service training to higher degree than their colleagues who do not have the same prerequisites. We might have expected a compensatory function of in-service training where those with fewer credits and fewer prerequisites (in terms of GPA) would earn more credits over time. Our results can be compared with a study by Smylie (1988) that found that teachers who perceived themselves as being most effective were the same ones most interested in learning new and more effective methods of teaching.

### Limitations and Further Research

In this article, we focused on the development of teachers’ knowledge in terms of university credits. The number of credits is an excellent quantitative measure that should be further investigated in relation to student achievement. However, it must be noted that this measure neither capture quality of in-service training nor all possible in-service training. Moreover, while we had access to a large sample of teachers, we cannot link teachers to students and cannot investigate the effects of the number of credits on student achievement. An option could be to aggregate teacher and student data to school-level and investigate schools’ average level of credits and relate this number to student achievement. We believe that future research should investigate this possibility. It would have been interesting to shed light on the credits in courses with focus on CK and PCK, respectively, but the large number of courses and teachers made this impossible within the scope of this article. For further research, however, we believe that a smaller sample of teachers could be selected for in-depth analysis, where their courses could be investigated in detail to highlight the prevalence of CK and PCK credits. In line with Shulman’s (1987) theory, teachers’ CK and PCK may improve the reading success of primary school students (Croninger et al., 2007), and to boost students’ mathematics in secondary school (Harris and Sass, 2011; Lachner and Nückles, 2016).

## Data Availability Statement

The data analyzed in this study is subject to the following licenses/restrictions: The data is derived from different registers with census data. Registers are typically accessible for researchers. All data was derived from Statistics Sweden (SCB) and analyzed within MONA (Microdata Online Access) which is Statistics Sweden’s platform for access to microdata. In MONA, users process data online without the microdata ever leaving Statistics Sweden. We combined micro level data in MONA from the teacher register and the Gothenburg Educational Longitudinal Database (GOLD). Requests to access these datasets should be directed to SJ, Stefan.johansson@gu.se.

## Author Contributions

SJ was the lead author and conducted data analysis and was responsible for writing up the final version of the manuscript. ÅH and TA made substantial contributions in conceptualizing the research and contributed to all parts of the study. All authors contributed to the article and approved the submitted version.

## Conflict of Interest

The authors declare that the research was conducted in the absence of any commercial or financial relationships that could be construed as a potential conflict of interest.

## Publisher’s Note

All claims expressed in this article are solely those of the authors and do not necessarily represent those of their affiliated organizations, or those of the publisher, the editors and the reviewers. Any product that may be evaluated in this article, or claim that may be made by its manufacturer, is not guaranteed or endorsed by the publisher.
